# 75 years of forensic profiling: A critical review

**DOI:** 10.1016/j.heliyon.2024.e39490

**Published:** 2024-10-18

**Authors:** Roberta Tittarelli, Sara Dagoli, Rossana Cecchi, Luigi Tonino Marsella, Francesco Saverio Romolo

**Affiliations:** a Department of Biomedicine and Prevention, Section of Legal Medicine, Social Security and Forensic Toxicology, University of Rome “Tor Vergata”, Via Montpellier 1, 00133, Rome, Italy; b Department of Medicine and Surgery, Legal Medicine, University of Parma, Viale Gramsci 14, 43126, Parma, Italy; c Department of Biochemical, Metabolic and Neural Sciences, Institute of Legal Medicine, University of Modena and Reggio Emilia, Modena, Italy; d Department of Law, University of Bergamo, Via Moroni 255, 24127, Bergamo, Italy

**Keywords:** Illicit drugs, Chemical warfare agents, Doping agents, Counterfeit medicines, Chemical profiling, Geo-profiling, Chemical attribution signatures (CAS)

## Abstract

The interest in characterization of drugs abused started in 1948 with the aim of determining the origin of opium. After 75 years there is still a great interest in this approach, called geo-profiling, chemical or forensic profiling in the following decades. Recently chemical attribution signatures (CAS) were proposed by the authors who studied “synthesis precursors and byproducts, impurities, degradation products, and metabolites in various biological matrices” of fentanyl. Forensic profiling evolved during these decades: new analytical approaches were tested and it was applied to more and more products, which threaten the health and security of citizens worldwide.

In substances of natural origins (e.g. opium, cannabis and cocaine), it is possible to exploit the great variability of both elements and organic chemical compounds and to study chemical compounds such as reagents and solvents, by-products, and cutting agents used in the production chain. Profiles can be used to classify products from different seizures into groups of similar samples (tactical intelligence) or to determine the origin of samples (strategic intelligence).

Chromatographic approaches coupled to mass spectrometry are very common to determine organic profiles, while elemental profiles are obtained by nuclear activation analysis, inductively coupled plasma mass spectrometry or ion beam analysis. A very important role in the field is played by isotope ratio analysis. Approaches to obtain forensic profiles are available also for chemical warfare agents, explosives, illegal medicines, doping agents, supplements, food. Chemometrics can be particularly useful to establish the authenticity of products and for the interpretation of large amount of forensic data. The future of forensic profiling is a challenge for forensic sciences. Organized crime is involved in the manufacturing of a large number of illegal products and forensic profiling is a very powerful tool to support the health of citizens and the administration of justice worldwide.

## Highlights

• Studies about forensic profiling of drugs abuse begun in 1948.
• Nowadays approaches for forensic profiling are available for many illegal products.
• Forensic profiling allows strategic intelligence, i.e. inference about the geographical origin.
• Forensic profiling allows tactical intelligence, i.e. inference about a common origin.

## 1 Introduction

An international program aimed at developing methods to determine the origin of opium by chemical and physical analytical tools was started in 1948 under the aegis of the United Nations. The results were described in 148 research papers, published over a period of 16 years under the serial heading "The assay, characterization, composition and origin of opium" [1]. The development of methods for the characterization of opium was discontinued in the late 1960s, when efforts began to focus on heroin, which was being increasingly abused [2,3].

In 1977 Henke published an article about the use of neutron activation analyses (NAA) of rare-earth elements in opium and cannabis and in the same year the United Nations International Drug Control Program organised a meeting of experts in Hong Kong to explore ways of 'profiling' or geo-locating heroin to support inferences about trafficking routes followed by organisation trading illicit drugs around the world [4]. Collins recently published an article describing the history of illicit drug profiling, beginning with the Hong Kong meeting [5].

The experience gained in the decades of analysis showed that in products of natural origins, such as cannabis, cocaine and opium, there is a great variability in chemical compositions due to impurities [6].

1. co-extracted from the raw plant materials (both elements and organic chemical compounds),
2. derived from laboratory processing (reagents and solvents used, by-products produced),
3. added at any point in the distribution chain (cutting agents).

The study of the profiles of such impurities obtained after chemical analysis of specimens of forensic interest, is called chemical or forensic profiling. Forensic profiling can support inferences about the similarity between specimens based on comparison of impurities. The comparison of the impurity profiles co-extracted from the seized raw plant materials with a set of samples of known geographical origin, can support inferences about the geographical origin specimen seized.

After the meeting in Hong Kong, the Commission on Narcotic Drugs, recognizing the need for an international strategy allowing characterization and profiling of impurities in narcotic drugs and psychotropic substances, requested the Executive Director of the United Nations International Drug Control Programme (UNDCP) to develop standard methods for the profiling/signature analysis in its resolution 1 (XXXIX) of 24 April 1996 [3]. The same Commission on Narcotic Drugs recognized the value of illicit drug characterization and profiling in supporting law enforcement intelligence-gathering and operational work and the international fight against illicit drugs with the resolution 47/5 of 19 March 2004 and with the resolution 50/9 of 16 March 2007.

Finally, the resolution 56/5 “Promoting the sharing of expertise in and knowledge on forensic drug profiling” acknowledged that forensic drug profiling and the measurement of external characteristics or those of the packaging materials, can be used to establish links between different drug seizures. These links can be combined with law enforcement intelligence to support the identification of targets or groups involved in drug trafficking, their methods, the chemicals used to manufacture drugs, and to contribute to a reduction of the illegal drug supply worldwide.

The forensic profiling of drugs requires global cooperation between forensic experts and law enforcement officers. It is important to share not only the expertise, knowledge, and best practices in this field, but also information on forensic profiling of drugs seized in clandestine laboratories and of relevant samples, especially if taken in connection with international investigations and for intelligence purposes.

In recent years, there has been an interest not only in analytical methods for forensic profiling of new substances, such as fentanyl, illegal pharmaceutical products, explosives and chemical warfare agents but also in chemometrics, allowing to promptly compare and interpret forensic data [7,8]. Both the reviews discussed in detail focused on the most common chemometric techniques in illicit drug profiling and we strongly suggest them to readers interested in this subject. Both the recent article by Collins [5] and the one by Ahmed et al. are very interesting, but they concentrated only on selected chemical compounds and listed a limited number of references [9]. Finally, the factors influencing the integration of illicit drug profiling in the forensic process were recently addressed [10].

The aim of this critical review is to provide an updated and comprehensive vision of the forensic profiling tools available for the main classes of illegal substances (e.g., heroin, cannabis, cocaine, and amphetamine-type stimulants), counterfeit medicines, dietary supplements, and chemical weapons, to support the fight against their clandestine production and global illicit trafficking based on the latest scientific news.

## 2 Forensic profiling of cannabis

Cannabis is one of the oldest known plants and its main active ingredient Δ-9-tetrahydrocannabinol (THC) is mainly responsible for the psychoactive effects [11]. Other related chemical compounds in cannabis include cannabidiol (CBD) and cannabinol (CBN) [12].

Cannabis products with high THC concentrations lead to an increased risk of addiction and mental health disorders, while it has been suggested that CBD may moderate the effects of THC [13].

The analyses of plants to determine their chemotype in term of increasing total psychoactive potential are verified by the THC/CBD ratio [14]. The inactive biosynthetic precursor tetrahydrocannabinolic acid (THCA) is also present in different amounts in cannabis plants [15]. Cannabis psychotropic effects, including anxiolysis and euphoria, results in its dangerous recreational use [16,17].

In Europe, the THC limit value for industrial hemp was first set at 0.5 % in 1984, then trimmed to 0.3 % in 1987, and further lowered to 0.2 % in 1999 to prevent the cultivation of illicit drug–type *Cannabis* in hemp fields [18]. The EU subsidies to hemp cultivation are granted upon the use of certified seeds from the varieties listed in the “Common Catalogue of Varieties of Agricultural Plant Species”, provided that THC content does not exceed 0.2 % [19,20]. Hemp imported in the EU must meet the same limit [21].

Quantification of cannabinoids in floral materials is, therefore, important to quantitatively evaluate its content [[22], [23], [24]].

Anxiety, depression, and psychotic symptoms can be amplified by a high THC content, which can as well increase the risk of addiction and adverse effects on the respiratory and cardiovascular systems in regular users [25]. Recent studies report increases in THC concentration in seized specimens of illegal cannabis for human consumption [26].

In 2020, Johnson et al., used a handheld Raman spectrometer for a survey of suspected drugs seized from several New Zealand music festivals [27].

Several countries in Europe adopted drug testing as a harm reduction strategy. The exchange of drug trends information within the Trans European Drug Information (TEDI) network conducted by European drug checking services, allows for monitoring and responses coordination [28].

In addition to the qualitative and quantitative analysis of Δ9-THC, and the identification and quantification of CBD and CBN, other methods allowing chemical-profiling of cannabis products are required [29,30].

These studies are challenging because of several factors from which depend the chemical composition and that cause a high heterogeneity and variability in the metabolites [31,32].

Osman et al., in 1985, used chromatographic patterns from headspace (HS) of the same cannabis samples and cannabis of different geographical origin, to explore the use of a retention index matrix (*r*-matrix) [33]. In the same year, a new approach was developed by Osman and Caddy to analyse cannabis samples after absorption of a wide range of volatile constituents (volatiles of cannabis resin) by Tenax, followed by gas chromatographic (GC) analysis [34].

In 2014, Zamengo et al., investigated 4000 samples of cannabis products between 2010 and 2012 [35]. The variation in THC content of each sample was very wide, ranging from 0.3 % to 31 % for cannabis resin and from 0.1 to 19 % for herbal cannabis. Over the past ten years, Δ9-THC concentration has increased dramatically going from 8.9 % in 2008 to 17.1 % in 2017.

Concentrations of THC, CBD and CBN of more than 12,000 cannabis samples were collected by Zamengo et al., in 2020 [36]: while CBD concentrations were found to decrease constantly over the study period, an opposite trend was observable with the median THC concentrations increasing from about 6% to 11 %.

The mean Δ9-THC/CBD ratio also rose substantially from 23 in 2008 to 104 in 2017 as reported in another study conducted by Chandra et al., using a validated method based on GC with a flame ionization detector (FID) [37].

The assessment of recent changes in the composition of seized cannabis resin was conducted also by Thomsen et al. [38], in Denmark. A dramatic increase in cannabis resin samples with high THC concentration was identified after 2011 along with the near disappearance of cannabis resin samples with medium and low THC concentration. Furthermore, THC/CBD concentration ratio has dramatically increased in Denmark.

Because the THC content is often higher than the levels of the other phytocannabinoids, an HPLC method was used to determine the content of nine cannabinoids: THC, cannabidiol, cannabigerol (CBG), the carboxylic acid precursors THC-A, CBD-A and CBG-A, as well as cannabichromene (CBC), cannabinol and tetrahydrocannabivarin (THC-V) by Swift et al. [39]. Trends concerning the use of high potency cannabis with very low CBD content seized by police in other countries are also reported in the paper.

A validated high-performance liquid chromatography-diode array detection (HPLC-DAD) method, allowing the simultaneous quantification of both the acidic and the neutral forms of THC, cannabidiol, and cannabinol was used by Boumrah et al. to analyse hashish samples [40].

Hädener et al. presented an HPLC-DAD method for quantitative analysis of major neutral and acidic cannabinoids in herbal cannabis and hashish [41]. Ambach et al., developed a method based on HPLC-DAD to quantitate THC, THCA-A, CBD, and CBN in confiscated products containing cannabis [42].

An important aspect to dwell on concerns the recreational or medical use of cannabis, requiring to distinguish recreational use of cannabis from intake of medicines containing cannabis chemical substances [43]. Cannabinoid profiles are needed to determine the use of medical cannabis, Sativex® or Dronabinol. In a study conducted by Scheunemann et al., a validated liquid chromatography-tandem mass spectrometry (LC-MS/MS) method was used to quantify 16 phytocannabinoids in specimens of Sativex® and Dronabinol from two different manufacturers [44]. Resultant cannabinoid profiles were compared, and markers were suggested. Acidic cannabinoids, cannabigerol and cannabinol occurred only in low amounts, and the presence of cannabichromene was considered characteristics of Sativex®. A specific cannabidiol/tetrahydrocannabinol ratio was also studied. Medical marijuana and seized cannabis were compared by principal component analysis (PCA). Several medical marijuana varieties were found to significantly differ from seized cannabis considering the contents of tetrahydrocannabinolic acid A, tetrahydrocannabivarinic acid, cannabidiolic and cannabidivarinic acids.

Mass spectrometric detectors hyphenated with typical separation techniques (i.e., liquid chromatography or gas chromatography) represent powerful approaches to study the features of cannabis specimens, improving the classification of cannabis varieties [[45], [46], [47], [48]]. Stempfer et al. used non-targeted LC-MS/MS to detect the compounds in seized cannabis [49]. One of the most-used analytical techniques for determination of phytocannabinoids remains gas chromatography/electron ionization-mass spectrometry (GC/EI-MS) [50].

Cássia Mariotti et al. used GC-MS associated with exploratory and discriminant analysis to study of cannabis seeds cultivated in greenhouses [51]. The chemical profiles of the specimens showed significant differences, probably because of their variety, light exposition, and age.

Sixty-eight samples of cannabis seeds from seizures performed by the Brazilian Federal Police were analysed by positive and negative electrospray ionization coupled to Fourier transform ion cyclotron resonance mass spectrometry (ESI (±)-FT-ICR MS and ESI(±) MS/MS) techniques by Borille et al. [52].

Cannabis seeds were analysed also by Tassi Borrile et al. [53], through NIR spectroscopy combined with chemometrics.

Slosse et al., in 2020 combined the cannabis profiles obtained by GC-MS with a statistical methodology to evaluate the presence of plantation variability [54]. The study of the predictive performance of the model supports the representativeness of the entire plantation information.

Cannabinoids composition characteristics were affected by factors subsequently investigated by Tipparat et al., using GC-FID to assist in the regulation criteria development of hemp cultivation in Thailand [55]. A GC method coupled with mass spectrometry (MS) was used by Ahmed et al., for the identification and the quantification of cannabinoids extracted from dried flowers of different *Cannabis* varieties [56].

Proton nuclear magnetic resonance (^1^H NMR) spectroscopy was also used to identify the major cannabinoids by de A Leite et al. [57].

Cannabis can be classified into two types: drug-type cannabis, worldwide abused, and fiber-type cannabis [58,59]. The differences in the sequences of tetrahydrocannabinolic acid synthase (THCAS) and cannabidiolic acid synthase (CBDAS) genes result in these two types. Yamamuro et al. developed a PCR-based method to distinguish between drug-type and fiber-type cannabis, by detecting differences in the sequences of *THCAS* and *CBDAS* [60].

With the use of a high-resolution melting (HRM) strategy combined with a barcoding marker, internal transcribed spacer (ITS), the genetic composition of seized *Cannabis* spp. in the south of Chile was determined by Solano et al. [61].

A study conducted by de Oliveira Pereira Ribeiro et al., had the aim of evaluating the effectiveness and efficiency of two short tandem repeats (STRs) multiplex systems previously proposed in ninety-four Brazil seized Cannabis sativa samples [62]. Principal coordinate analyses (PCoA), forensic parameters, and genetic structure analysis were executed. The two panels were both effective when it came to individualizing and origin discriminating all samples. A clear genetic distinction among samples according to its origin is shown by the PCoA.

A better understanding of marijuana distribution networks can be gained thanks to a stable isotope ratio analysis of marijuana seizures. Geographic region-of-origin and growth environment for marijuana can be predicted using hydrogen and carbon isotope ratios according to the stable isotope-based models described by Hurley et al. [63]. Furthermore, the delta (13)C and delta (15)N of 508 domestic samples from known U.S.A. counties were analysed by West et al. [64].

Verifying the possible presence of adulterants in cannabis products presents an important challenge [65]. In 2021, Oomen et al. evaluated Cannabis products suspected of adulteration that were analysed for the presence of MDMB-4en-PINACA by nine services in eight countries [66]. The adulteration of cannabis with synthetic cannabinoid receptor agonists represents a new phenomenon carrying risks for people who use it [67]. Recently, Antoine et al. used NAA and multivariate analysis to understand where the Jamaican *Cannabis sativa* L. comes from Ref. [68].

In Table 1, a summary of the separation and identification techniques for *Cannabis* profiling is reported.

**Table 1:** Techniques employed for *Cannabis* profiling.

Analytical methods	Herbal Cannabis	Cannabis resin	Seeds	Pharmaceuticals/Commercial products	Adulterants	References
GC-FID	X	X				Chandra et al., 2019 [37]
	X					Tipparat et al., 2012 [55]
	X	X				Zamengo et al., 2014 [35]
	X	X				Zamengo et al., 2020 [36]
		X				Rømer Thomsen et al., 2019 [38]
	X					Cascini et al., 2019 [58]
	X					André et al., 2020 [59]
FTIR	X					Johnson et al., 2020 [27]
FT-ICR MS			X			Borille et al., 2017 [52]
IRMS	X					West et al., 2009 [64]
GC-MS	X					Osman et al., 1985 [34]
	X					André et al., 2020 [59]
	X	X				Zamengo et al., 2014 [35]
	X					Johnson et al., 2020 [27]
		X				Osman et al., 1985 [34]
	X	X				Zamengo et al., 2014 [35]
			X			Mariotti et al., 2016 [51]
	X					Mhando et al., 2023 [45]
	X					Slosse et al., 2023 [47]
	X					Slosse et al., 2020 [54]
	X					Ahmed et al., 2021 [56]
		X				Johnson et al., 2020 [50]
HS-GC/MS	X					Osman et al., 1985 [33]
LC-MS	X					Cascini et al., 2019 [58]
	X					Stempfer et al., 2021 [49]
				X		Scheunemann et al., 2021 [44]
	X					Swift et al., 2013 [39]
HPLC-MS	X					Andrè et al., 2020 [59]
HPLC-DAD	X					Boumrah et al., 2020 [40]
		X				Hädener et al., 2019 [41]
		X				Ambach et al., 2014 [42]
NMR	X					A. Leite et al., 2018 [57]
					X	Meehan-Atrash et al., 2020 [65]
TC/EA-IRMS	X					Hurley et al., 2010 [63]
NIR			X			Borille et al., 2017 [53]
Raman spectroscopy	X					Johnson et al., 2020 [27]
Genetic analysis	X					Roman et al., 2020; Cascini et al., 2019; Yamamuro et al., 2021^43,58,60–62^
NAA	X					Antoine et al., 2022 [68]

Legend:**GC-FID**: Gas Chromatography With Flame-Ionization Detector; **IRMS**: Isotope Ratio Mass Spectrometry; **GC-MS:** Gas Chromatography Mass Spectrometry; **HS-GC/MS**: Headspace-Gas Chromatography Mass Spectrometry; **FTIR:** Fourier transform infrared spectroscopy; **LC-MS:** Liquid Chromatography Mass Spectrometry; **HPLC:** High Performance Liquid Chromatography; **HPLC –MS:** High Performance Liquid Chromatography Mass Spectrometry; **HPLC-DAD**: High Performance Liquid Chromatography-diode array detector; **NMR:** Nuclear Magnetic Resonance; **NIR:** Near Infrared Reflectance; **NAA**: Neutron Activation Analysis.

## 3 Forensic profiling of heroin

According to the data reported by the United Nations on Drugs and Crime (UNODC) in the World Drug Report 2023, world consumption of opiates is estimated to be approximately 31.5 million, mainly heroin users [69]. Most of the heroin produced worldwide comes from Afghan opium whereas the rest is yielded in Myanmar and the Lao People's Democratic Republic. Most of heroin produced in Afghanistan (approximately 375 tons) is trafficked worldwide via the neighbouring countries. The Balkan and northern routes are the main heroin trafficking passages between Afghanistan and the Russian Federation. The turnover of this market amounts to $ 13 billion per year [70]. These data clearly show that the fight against heroin illicit trade would benefit from the development of new forensic methodologies aimed at the determination of the geographical origin of the seized samples and at trafficking heroin networks dismantlement [71].

The study of co-extracted impurities from the raw plant materials (both elements and organic chemical compounds) of opium and heroin has a long history. Heroin manufacturing also results in the production of trace amounts of acidic and neutral impurities during the reaction of acetic anhydride with morphine, codeine, thebaine, papaverine, noscapine, norlaudanosine, reticuline, narceine and other opium alkaloids [[72], [73], [74], [75]]. The presence or absence and the ratio of these impurities are a direct result of the manufacturing process used to obtain a batch of heroin from opium. Therefore, the heroin profiling also involves the analysis of major alkaloids, basic impurities, as well as occluded solvents and trace metals by various chromatographic and/or spectrometric techniques, to provide valuable information about the origin of the opium and the heroin production process [[76], [77], [78], [79]].

In 1970 Curry and Patterson performed qualitative analyses to detect the presence of heroin and adulterants (e.g., caffeine, barbiturates, quinine) in seized powders, using infrared spectroscopy (IR), thin-layer chromatography (TLC) and gas-liquid chromatography (GLC) [80,81].

Huizer H. in 1983 developed a TLC and a high-pressure liquid chromatographic (HPLC) method to detect the presence of O3-monoacetylmorphine in illicit heroin samples and its feasible formation during the manufacturing process of heroin [82].

Due to the magnitude of the problem and the importance of chemical analytical approach to heroin profiling, many organizations performed research in the field of drug profiling over many years [[83], [84], [85]]. So, other analytical methods were developed by several authors to perform chemical analyses of heroin components for samples comparison: in particular, gas chromatographic analysis was employed for the identification of heroin and its geographic origin determination based on of the acid and neutral byproducts [86,87].

Packed-columns GLC coupled with a FID or an electron capture detector (ECD) were used for the identification of trace amounts components [[88], [89], [90], [91], [92], [93], [94]].

Besacier et al., in 1997, developed an innovative methodology based on a three-step procedure involving GC-FID and gas chromatograph-isotope ratio mass spectrometer (GC-IRMS) technologies for the comparative chemical analyses of seized heroin samples [95]. Ehleringer et al. analysed the carbon and nitrogen stable isotope ratios in 76 heroin and 28 cocaine samples demonstrating that results strongly correlated with geographic regions of origin [96]. Casale et al. later studied the possible changes of ^13^C and ^15^N during the acetylation of morphine to heroin and found that the heroin produced had more negative δ^13^C values compared the morphine of origin while after deacetylation of heroin, the resulting morphine had the same δ^13^C of the original morphine [97]. According to Thompson et al., carbon and nitrogen isotope ratio enhance the ability to geo-source heroin when used with traditional chemical profiling approaches [98].

In 2000 Strömberg et al. presented a retrospective study for the comparison of south west Asian heroin, consisting in the development of a GC-FID method together with a computerized database, and a retrieval system to storage the chemical profiles of the seized heroin samples in an updated library facilitating in this way their comparison [99].

The application of capillary gas chromatography columns led to a better resolution of the complex mixtures and allowed the analyses of both derivatized and underivatized compounds for comparison or profiling purposes [[100], [101], [102], [103]].

Since the early 2000s analytical methods based on GC and LC [104,105] coupled with mass spectrometry were successfully applied to opioids analysis with good sensitivity and high reproducibility, to improve the detection of the single components and to better identify unknown components [[106], [107], [108], [109], [110], [111]].

In 2001 Myors et al., developed a GC-MS procedure for the detection of over 649 organic constituents together with a statistical method for data analysis to fingerprint heroin samples and to predict their geographical origin [112].

A purge and trap GC-MS and a static headspace GC-MS methods were developed in 2006 by Collins et al., for the detection of acid/neutral manufacturing by-products and the identification of solvents traces in heroin specimens [113,114].

El-Haj et al. developed a GC-MS method for the identification of mannitol hexaacetate (MHA) as adulterant in some brown seized samples in which diacetylmorphine was not detected. MHA is formed by transacetylation of mannitol with diacetylmorphine: its presence is linked to an addition of mannitol in the early stages of heroin production, so it represents an important marker of clandestine drug production areas [115].

Morello et al., in 2010 described a new procedure to isolate and identify trace level of acidic and neutral impurities by programmed temperature vaporizing injector-gas chromatography-mass spectrometry (PTV-GC-MS), analyzing over 500 heroin samples and succeeding also in the classification of several diluents and adulterants as caffeine, acetaminophen, phenacetin and diltiazem [72].

Capillary electrophoresis (CE) could be used for the determination of drug-related impurities [116]. Micellar eletrokinetic capillary chromatography (MECC) coupled with an UV detector was applied by Altria et al. and Walker et al. for the detection of primary alkaloids, adulterants, and drug-related impurities [117,118], whereas a laser induced fluorescence detector was employed by Weinberg et al. and Lurie et al. for the identification of neutral and acidic impurities in heroin samples [119,120].

Toske et al., in 2006, revealed the presence of basic alkaloid impurities (e.g., acetylcodeine, noscapine, papaverine, etc.) in seized heroin samples by HPLC and CE [121]. The authors performed also a semiquantitative analysis of trace level acidic and neutral impurities, originated from the reaction of morphine and alkaloids with hot anhydride, originally using GC-FID technology and later developing a GC-MS method. Furthermore, the authors performed an analysis of occluded solvents of the crystal matrix by GC-MS headspace technology to detect the solvents used to manufacture heroin [75].

Several authors reported the use of HPLC system with UV detection in the reversed phase mode, for the comparative analysis of illicit heroin [122,123]. In 1984 Lurie et al. used HPLC coupled with UV detector to detect acidic and neutral acetylated rearrangement products of opium alkaloids [124]. Fluorescent spectrophotometric and electrochemical detectors were also used by several authors for heroin samples comparison [125].

To obtain a higher selectivity, Hays et al. performed the quantitation of adulterants in illicit heroin samples by reversed phase HPLC using a photo-diode array and a fluorescence detector [126].

In 2002 Dams et al., presented a fast LC-MS analysis interfaced with a sonic spray ionization (SS) to simultaneously detect several opium alkaloids in heroin impurity profiling [127]. The authors highlighted that although thermospray (TS) [128], electrospray (ES) [129], pneumatically assisted electrospray or ion spray (IS) [130] and atmospheric pressure chemical ionization (APCI) [131] interfaces were successfully applied for the quantification of opiates and their metabolites, the SS ionization had a high separation efficiency and better performances in ion formation efficiency compared to the ion spray source.

Notwithstanding the advantages introduced by LC-MS, emerging analytical techniques, such as ultra-high-pressure liquid chromatography (UHPLC), could offer better chromatographic performance in the detection of heroin impurities [132,133]. Lurie et al., described the feasibility of ultra-performance liquid chromatography tandem-mass spectrometry (UPLC-MS/MS) for heroin profiling. In 2008 the authors were able to detect at levels as low as 10^−6^ % w/w basic impurities (morphine, codeine, noscapine, papaverine, reticuline, narceine, codamine, laudanidine, cryptopine, laudanosine, norlaudanosine) and neutral impurities as N,3,6-triacetylnormorphine, N-acetylnorcodeine, N-acetylnornarcotine, 3,6-dimethoxy-4-acetyloxy-5-[2-(N-methylacetamido)]-ethylphenanthrene, and cis-n-acetylanhydronornarceine [134]. Later, in 2013, the same group reported a methodology for the analytical identification and ratios determination of basic impurities detected in seized heroin as a percentage equal to 0.02 %, using UHPLC [76].

Liu et al., in 2015, developed a new acidic and neutral manufacturing impurity profiling method using an ultra-performance liquid chromatography–quadrupole-time of flight mass spectrometry (UPLC–Q-TOF). The high resolution, mass accuracy and high sensitivity of this technique allowed them to identify 19 significant heroin impurities able to discriminate the geographical origin of heroin samples (“Golden Triangle” and “Golden Crescent”) [73].

Liu et al., in 2016, developed and validated a new rapid and sensitive UPLC-Q-TOF method for the determination of five principal and 14 minor opium alkaloids in gum opium. The new analytical method together with the elaboration of a data set of principal and minor opium alkaloids based on the characteristics of “Myanmar” and “Afghanistan” opium samples allowed the construction of a model for the classification of opium in relationship to its geographical origin [135].

Bourmaud et al. studied results of analysis carried out in 2019 and 2020 by GC–MS, HPLC-UV and LC-Q-TOF and X-ray fluorescence analyses on heroin and cocaine specimens. The researchers suggested that both the elaboration of drug and harm prevention and law enforcement strategies should consider cutting processes, adulteration practices and variability of quality and purity of street drugs and further international studies on this topic are needed [136].

The classification of heroin samples based on elemental profiling by inductive coupled plasma mass spectrometry (ICP-MS) is also reported in literature [[137], [138], [139]]. In their study Liu et al., presented a new heroin profiling strategy by ICP-MS for heroin identification from ‘‘Golden Triangle’’ and ‘‘Golden Crescent’’ based on 19 inorganic elements fingerprinting (Ag, As, Ba, Cd, Co, Cr, Cu, Mn, Mo, Ni, P, Pb, Se, Sb, Th, Tl, U, V and Zn). The presence or the absence of these elements, introduced in different phases of heroin clandestine manufacturing, and their ratios could be helpful for a comparative analysis focused on the origin geographical area determination [140].

Before the advent of ICP-MS, NAA has been used to analyse trace metal elements for long time, determining important information about the presence of trace elements and their concentrations in drug samples [141].

Zhang et al. studied illicit heroin by NAA and grouped sixty-two analysed heroin specimens in two clusters associated to their geographical origins [142]. Ebrahimi et al. analysed opium, hashish and ecstasy pills by both NAA and Particle Induced X-ray Emission (PIXE) [143].

The latest approach to illicit drug profiling is based on cutting agents (adulterants and diluents) identification by Isotopic Ratio Monitoring by Mass Spectrometry (IRM-MS) and on heroin and 6-MAM recognition at a basic pH by electrochemical sensors [144]. Joubert and al. in 2020 reported how both ^13^C NMR spectrometry and ^13^C, ^15^N MS might provide useful information to link seized caffeine and paracetamol to their origin [145]. IRM by ^13^C NMR (IRM-^13^C NMR) offers additional benefits over IRM-MS to determine a detailed isotopic profile, to better distinguish different caffeine and paracetamol batches. In Table 2 the most widespread techniques for heroin profiling are reported.

**Table 2:** Techniques employed for heroin profiling.

Analytical methods	Trace elements	Alkaloids and other components	Solvents	Adulterants and cutting agents	Organic Impurities and by-products	Isotope ratio	References
IR				X			Curry et al., 1970 [80]
TLC				X			Curry et al., 1970 [80]
					X		Huizer, 1983 [82]
GC-FID		X		X			Law et al., 1983 [90]
		X					Barnfield et al., 1988 [93]
		X					Gough et al., 1981 [89]
		X					Law et al., 1983 [90]
		X					Chow et al., 1983 [106]
		X					Besacier et al., 1997 [95]
					X		Strömberg et al., 2000 [99]
					X		Esseiva et al., 2003 [109]
		X					Casale et al., 2017 [86]
					X		Toske et al., 2006 [75]
GC-ECD		X					Moore, 1978 [92]
		X					Barnfield et al., 1988 [93]
		X					Gough et al., 1981 [89]
					X		Moore, 1983 [101]
Capillary GC					X		Allen et al., 1984 [74]
		X					Gloger et al., 1983 [103]
					X		Moore, 1984 [102]
Capillary GC-MS					X		Allen et al., 1984 [74]
		X			X		Neumann 1994 [78]
GC-IRMS						X	Besacier et al., 1997 [95]
GC-MS					X		Morello et al., 2010 [72]
					X		Toske et al., 2006 [75]
		X					Casale et al., 2017 [86]
					X		Myors et al., 2001 [112]
					X		Collins et al., 2006 [113]
				X			El-Haj et al., 2004 [115]
					X		Morello et al., 2010 [72]
					X		Toske et al., 2006 [75]
PTV-GC/MS		X					Casale et al., 2017 [86]
HS-GC/MS			X				Toske et al., 2006 [75]
					X		Collins et al., 2006 [113]
HRCGC		X					Neumann et al., 1982 [100]
GC-HRMS					X		Toske et al., 2006 [75]
SHS-GC-MS			X				Collins et al., 2007 [114]
GLC				X			Curry et al., 1970 [80]
		X					Narayanaswami et al., 1979 [94]
		X					O'Neil et al., 1984 [107]
GLC-MS		X					Chow et al., 1983 [106]
CE		X		X	X		Lurie et al., 2004 [121]
		X					Toske et al., 2006 [75]
					X		Altria, 1996 [117]
					X		Lurie et al., 1995 [120]
		X					Collins et al., 2006 [113]
MECC		X					Weinberger et al., 1991 [119]
		X		X	X		Walker et al., 1995 [118]
LC		X					Collins et al., 2006 [113]
LC-MS		X					Pichini et al., 1999 [104]
					X		Dams et al., 2002 [127]
		X					Zuccaro et al., 1997 [130]
		X					Bogusz et al., 1997 [131]
HPLC					X		Huizer, 1983 [82]
		X		X			Johnston et al., 1998 [85]
		X		X			Law et al., 1983 [90]
		X					Chow et al., 1983 [106]
		X					O'Neil et al., 1984 [107]
					X		Lurie et al., 1984 [124]
					X		Law et al., 1984 [133]
		X					Toske et al., 2006 [75]
Reversed phase HPLC					X		Lurie et al., 1987 [77]
				X	X		Kaa, 1994 [122]
		X					Kaa, 1991 [123]
				X			Hays et al., 1991 [126]
HPLC-MS					X		Toske et al., 2006 [75]
					X		Lurie et al., 2013 [76]
		X					Pacifici et al., 1995 [129]
UPLC-MS/MS		X					Lurie et al., 2008 [134]
UHPLC-MS/MS		X					Debrus et al., 2011 [132]
UPLC-Q-TOF					X		Liu et al., 2015 [73]
		X					Liu et al., 2015 [73]
ICP-MS	X						Wells et al., 1995 [137]
	X						Myors et al., 1998 [138]
	X						Chan et al., 2013 [139]
	X						Liu et al., 2014 [140]
NMR					X		Toske et al., 2006 [75]
		X					Casale et al., 2017 [86]
					X	X	Joubert et al., 2020 [145]
NAA	X						Tuckerman et al., 1964 [141]
	X						Zhang et al., 2004 [142]
	X						Fakhar et al., 2012 [143]
PIXE	X						Fakhar et al., 2012 [143]
Electrochemical Profiling		X					Felipe Montiel et al., 2022 [144]

Legend:**IR**: Infra-Red Spectroscopy; **TLC**: Thin-Layer Chromatography; **GC-FID**: Gas Chromatography With Flame-Ionization Detector; **GC-ECD:** Gas Chromatography With Electron Capture Detector; **Capillary GC:** Capillary Gas Chromatography; **Capillary GC-MS:** Capillary Gas Chromatography Mass Spectrometry; **GC-IRMS**: Gas Chromatograph-Isotope Ratio Mass Spectrometry; **GC-MS:** Gas Chromatography Mass Spectrometry; **PTV-GC/MS:** Programmable Temperature Vaporizer Gas Chromatography Mass Spectrometry; **HS-GC/MS**: Headspace-Gas Chromatography Mass Spectrometry; **HRCGC:** High-Resolution Capillary Gas Chromatography; **GC-HRMS:** Gas Chromatography/High-Resolution Mass Spectrometry; **SHS-GC-MS**: Static Headspace-Gas Chromatography-Mass Spectrometry; **GLC**: Gas-Liquid Chromatography; **GLC-MS:** Gas-Liquid Chromatography Mass Spectrometry; **CE:** Capillary Electrophoresis; **MECC:** Micellar Electrokinetic Capillary Chromatography; **LC-MS:** Liquid Chromatography Mass Spectrometry; **HPLC:** High Performance Liquid Chromatography; **HPLC –MS:** High Performance Liquid Chromatography Mass Spectrometry; **UPLC-MS/MS:** Ultra-Performance Liquid Chromatography-Tandem Mass Spectrometry**; UHPLC-MS/MS:** Ultra-High-Performance Liquid Chromatography-Tandem Mass Spectrometry**; UPLC-Q-TOF:** Ultra-High Performance Liquid Chromatography-Quadrupole Time-Of-Flight Mass Spectrometry**; ICP-MS:** Inductively Coupled Plasma Mass Spectrometry; **NMR:** Nuclear Magnetic Resonance; **NAA**: Neutron Activation Analysis; **PIXE** Proton Induced X-Ray Emission.

## 4 Forensic profiling of cocaine

Cocaine is a tropane alkaloid and stimulant drug obtained primarily from the leaves of two coca species. It is one of the most widely used illicit drug worldwide. According to the data reported by UNODC in the World Drug Report 2023, the total cocaine production increased every year between 2014 and 2021, when it reached 2304 tons. In that year 22 million people had used cocaine [69].

Also in 2021, the cultivation of coca in Colombia topped a historical high level, with 5 new coca production enclaves identified, to be added to the 9 already known in the past years [146]. Coca leaf cultivation recently increased also in Bolivia and Perù between 2020 and 2021 [147].

The review by Collins [5] and the one by Ahmed et al. [9] refer several studies about profiling of impurities in cocaine, beginning with the paper by Lukaszewski T, Jeffries published in 1980 [148]. In 1988 Lebelle et al. described a cocaine examination laboratory system. Thanks to capillary column gas chromatography and an ion trap detector the separation and identification of the different components in the exhibits were achieved. Further examination and quantification of the compounds was obtained using two HPLC systems [149]. After a few years, Lebelle used the combination of HPLC and GC with both a flame ionization detector and a mass spectrometer to determine the minor components, which are present in illicit uncut cocaine [150].

Since 1990 the attention was focused on profiling a group of minor alkaloids called isomeric truxillines, which could be found in all illicit cocaine specimens [151]. In 1992 Janzen et al. described a method for illicit cocaine samples comparison analysis. The data were obtained by capillary GC using a nitrogen phosphorus detector. The area ratios of the alkaloids tropacocaine, norcocaine, cis-cinnamoylcocaine and trans-cinnamoylcocaine to cocaine were determined for each sample [152].

Geographically sourcing cocaine by chemical profiling needs the centralised collection of data in one database [153], where should be stored as many analytical results as possible obtained from coca leaves cultivated in South America or in greenhouse [154]. Not only the variety of coca used for production could be determined based on the alkaloid content of cocaine, but also the probable region of growth [[155], [156], [157], [158]].

Impurity profiles of seized cocaine enable the classification of material from different seizures into groups of similar samples (tactical intelligence), and to identify the origin of samples (strategic intelligence). Such information can be used for evidential purposes (judicial, court) or it can be considered as a source of intelligence to identify samples that may have a common origin or history [2,[159], [160], [161]].

The sourcing of cocaine origin was significantly improved thanks to the use of trace alkaloid data combined with isotope ratios or purified cocaine by Ehleringer et al., who demonstrated that the study of stable isotope ratios of carbon and nitrogen allow the determination of the country of origin for the principal coca-growing regions in South America [162]. In 2007 Sewenig et al. published an article about the analysis of 132 cocaine specimens from a big seizure occurred in Germany in 2002, carried out to determine the δ^13^C and δ^15^N results [163].

Illicit processing methods leave stable isotopes present in cocaine unaffected and this allow inferences about the environment in which the coca was cultivated, based on purified cocaine specimens analysed after seizures. The study of both natural variations in alkaloids and stable isotopes can therefore be used to classify cocaine based on the country of origin. The discriminatory ability of existing geo-sourcing analysis was later improved by adding to the analytical approach two more stable isotopes: ^2^H and ^18^O). Moreover, chemometrics based multivariate statistical analysis and exploratory spatial data analysis (ESDA) were developed to obtain simultaneous evaluation of all data collected to show the known growing regions [164]. This approach permitted to accurately determine sub-regional classifications of specimens of cocaine. Based on results from more than 500 samples of coca leaf from 19 coca-growing regions were analysed. Cocaine was extracted, purified, and, subsequently, analysed for total alkaloid content. Moreover, the isotopes δ^13^C, δ^15^N, δ^2^H, and δ^18^O were measured in each collected coca leaf sample. The data obtained from each sample were associated not only to the general sub-regional location, but also with the geographical coordinates of the originating field and each variable was visualised across a very large area. According to the results each alkaloid (tropacocaine, trimethoxycocaine and truxillines) was present with a different percentage depending on the origin of the region, although different percentages were sometimes individuated within the same region. The overlap complicates sourcing any specimen to a sub-region if only trace alkaloid data are taken into consideration. Isotopic signature observed in cocaine reflects environmental effects on the coca plant. For example, effects can be related to temperature and CO_2_ differences due to altitude gradients [165,166]. Moreover, the δ^15^N data from cocaine samples throughout South America relate with the precipitations and soil type. The enrichment of ^15^N in tropical environments can be related to increased nitrogen availability [167]. It has been demonstrated that deuterium and oxygen isotope ratios in cocaine are directly influenced by humidity conditions and source water. Based on the work by Mallette et al. it was also possible to find new regions within South America where coca cultivation began [164].

The isotope ratio approach was not tested only on cocaine specimens: Galimov et al. proposed gas chromatography/combustion/mass spectrometry (GC-C-MS) and elemental analysis/mass spectrometry (EA-MS) techniques to determine δ^1^C and δ^15^N data from heroin, morphine, cocaine and hemp leaves, specimens [168].

The Australian Illicit Drug Intelligence Program focused on 4 signatures: signature 1, based on the quantification of 14 alkaloids, particularly tropacocaine, 3′,4′,5′-trimethoxycocaine and the cinnamoylcocaines; signature 2, obtained using the truxillines data; signature 3, based on occluded solvents; signature 4, determined thanks to the ^13^C/^12^C and ^15^N/^14^N isotope ratios measurements [114].

Illicit cocaine seizures are still compared using alkaloid profiles obtained by GC-MS to infer about a possible common origin [160,169,170].

Another approach proposed to obtain chemical profiles of cocaine samples allows the profiling of residual solvents of the samples using headspace gas chromatography-mass spectrometry (GC-MS), as demonstrated by Monfreda et al. [171] and Nielsen et al. [172]. Nielsen et al. compared the profiles of alkaloids and of residual solvent obtained from 198 cocaine bricks [173].

Maldaner et al. recently developed and validated a method for minor alkaloids quantification in cocaine using the flame ionization detector after derivatisation [174]. Gas chromatography coupled with mass spectrometry was utilised for residual solvent assessment in seized cocaine and heroin by Cabarcos et al. [175].

In more recent year, HPLC was also tested in cocaine profiling. A UHPLC-QTOF-MS method was developed and validated for the first time in China, by Liu et al., to analyse simultaneously 14 cocaine alkaloids and 5 main adulterants [176]. The comparison of data from seized cocaine specimens obtained by UHPLC-TOF-MS comprehensive drug screening, was performed by Carby-Robinson et al., who discovered 53 potential markers for chemical profiling of cocaine using a non-targeted approach [177].

The study of ’cutting’ or additive agents in cocaine, like benzocaine (BZC), is the base for police analysts to obtain information about transformation steps of bulk drugs into street samples. Detection of organic impurities in cocaine samples seized in China was obtained by direct analysis in real time (DART) high-resolution mass spectrometry (HRMS) [178].

Electropolymerized molecularly imprinted polymers (e-MIPs) on portable screen-printed carbon electrodes (SPCEs) were also developed in a study for BZC determination conducted by Grothe et al. [179].

An infrared Spectroscopy strategy with Fourier Transformed and Attenuated Total Reflectance-based strategy (ATR-FTIR) associated with chemometrics was proposed to identify the chemical “fingerprint” of cocaine samples [180].

A portable NIR spectrophotometer was tested by federal police of Brazil to characterize seized cocaine specimens *in situ*. NIR/chemometrics based analytical protocol proved useful for the purpose of reliably screen the seized samples [181].

Pagano et al. proposed a strategy based on (1)H NMR analysis coupled with multivariate analysis to determine what they call the chemical “fingerprint” of cocaine specimens, allowing to link them [182].

Finally, it is important to consider the inorganic element profiling of cocaine. This was performed, for example, at the National Narcotics Laboratory in China by ICP-MS for analysing simultaneously 26 inorganic elements in 183 cocaine specimens [183]. Amorim et al. recently published a paper reporting both ICP-MS and inductively coupled plasma–optical emission spectrometry (ICP-OES) results obtained with cocaine specimens seized in three different areas in Brazil [184].

In 2022 Laposchan et al. published an extensive review of chemical substances found in cocaine specimen seized based on retrospective data mining [185]. The incidence and concentration on impurities and adulterants were studied to provide forensic intelligence. Data from the forensic laboratory of the Dutch National Police in Amsterdam were used to preliminarily assess that the detection of chemical substances selected as target by the researchers is feasible. Small changes to the analytical method improve the detectability of target analytes. A summary of the main separation and identification techniques for cocaine profiling is reported in Table 3.

**Table 3:** Techniques for cocaine profiling.

Analytical methods	Cocaine plants	Alkaloids	Cocaine samples	Adulterants and cutting agents	Organic and inorganic impurities and by-products	References
IR		X				Moore et al., 1995 [157]
TLC		X				Ensing et al., 1991 [151]
GC-FID		X				LeBelle et al., 1991 [150]
		X				Moore et al., 1994 [154]
		X			X	Moore et al., 1994 [155]
		X				Maldaner et al., 2021 [174]
Capillary GC		X				Lurie et al., 1990 [149]
		X				Janzen et al., 1992 [152]
GC-MS		X				LeBelle et al., 1991 [150]
		X				Moore et al., 1995 [157]
		X			X	Moore et al., 1998 [158]
				X		Broséus et al., 2015 [160]
			X			Souza et al., 2016 [169]
					X	Laposchan et al., 2022 [185]
		X				Villesen et al., 2017 [170]
		X				Monfreda et al., 2015 [171]
		X			X	Moore et al., 1994 [155]
		X				Stride Nielsen et al., 2016 [172]
		X			X	Stride Nielsen et al., 2017 [173]
					X	Cabarcos et al., 2016 [175]
DART-HRMS					X	Cui et al., 2019 [178]
HPLC		X				Lurie et al., 1990 [149]
		X			X	Moore et al., 1994 [155]
		X				Moore et al., 1995 [157]
HPLC-FID		X				LeBelle et al., 1991 [150]
UPLC-Q-TOF		X			X	Liu et al., 2017 [176]
		X			X	Carby‐Robinson et al., 2022 [177]
ICP-MS					X	Liu et al., 2017 [183]
					X	Amorim et al., 2021 [184]
		X				Lurie et al., 1990 [149]
			X			Pagano et al., 2013 [182]
Raman spectroscopy		X				Moore et al., 1995 [157]
GC-IRMS		X				Ehleringer et al., 2000 [162]
		X				Sewenig et al., 2007 [163]
	X					Mallette et al., 2016 [164]
	X					Körner et al., 1988 [165]
	X					Körner et al., 1991 [166]
	X					Martinelli et al., 1999 [167]
	X				X	Galimov et al., 2005 [168]
		X				Collins et al., 2007 [114]
FTIR		X				Monfreda et al., 2015 [171]
		X				Grothe et al., 2021 [179]
ATR-FTIR			X			Materazzi et al., 2017 [180]
NIR Spectrophotometer			X			Hespanhol et al., 2019 [181]

Legend:**IR**: Infra-Red Spectroscopy; **TLC**: Thin-Layer Chromatography; **GC-FID**: Gas Chromatography With Flame-Ionization Detector; **Capillary GC:** Capillary Gas Chromatography; **GC-MS:** Gas Chromatography Mass Spectrometry; **HPLC:** High Performance Liquid Chromatography; **UPLC-Q-TOF:** Ultra-High Performance Liquid Chromatography-Quadrupole Time-Of-Flight Mass Spectrometry**; ICP-MS:** Inductively Coupled Plasma Mass Spectrometry; **NMR:** Nuclear Magnetic Resonance; **DART-HRMS:** direct analysis in real time coupled with high-resolution mass spectrometry; **HPLC-FID:** High Performance Liquid Chromatography-Flame-Ionization Detector; **NIR Spectrophotometer:** near-infrared spectrophotometer; **FTIR:** fourier transform spectroscopy; **GC-IRMS:** Gas Chromatography- Isotope Ratio Mass Spectrometry; **ATR-FTIR:** infrared spectroscopy with fourier transformed and attenuated total reflectance.

## 5 Forensic profiling of synthetic illicit drugs of abuse

### 5.1 Amphetamine and amphetamine-type substances (ATS)

Characterisation of impurities were used also with synthetic illicit drugs, such as amphetamine and amphetamine-type substances (ATS), since the seventies of the past century. The aim was to establish the synthetic route and to evaluate the similarities between seized specimens [186], with the first review about impurities in amphetamine, mainly analysed at that time by GC, TLC, MS and NMR, published in 1981 [187].

The General Assembly of the UN adopted an “Action Plan against Illicit Manufacture, Trafficking and Abuse of Amphetamine-type Stimulants and Their Precursors” at the plenary meeting on 10 June 1998, recommending the development drug signature analysis and profiling [188].

The World Drug Report 2023 reports that 36 million people are the estimated amphetamines users and 20 million the users of substances similar to “ecstasy”. Seizures of “captagon”, an illicit product containing amphetamine often found in the Near and Middle East, reached in 2021 the record of 86 tons of amphetamine-based material. Methamphetamine is the primary drug reported by people beginning a treatment for drugs of abuse in East and South-East Asia [69].

ATS are often sold as pills or tablets and their physical examination can provide connections between seizures based on general feature (size, shape, colour) and imperfections related to the tool used to manufacture them [9].

The Drug Enforcement Administration's (DEA) started the Methamphetamine Profiling Program (MPP) in 1997. Analysis where initially carried out by GC-FID to determine the percentage of amphetamine and methamphetamine. HS analysis by GC-MS were used to determine trace solvents and ICP-MS analysis were carried out for elemental profiling [189]. Based on elemental profiles it is possible to demonstrate the use of specific catalysts (e.g., platinum oxide, palladium chloride or Raney Nickel) or reagents (e.g., sodium borohydride, mercuric chloride, and red phosphorus).

In 1997 the Federal Government of Australia established the “National Heroin Signature Program” and by 2002 the program was expanded to include ATS and changed its name to the “Australian Illicit Drug Intelligence Program” (AIDIP) [190]. The program focused on 2 signatures for MDMA or methylamphetamine. ATS Signature 1 aims to identify manufacturing by-products by liquid–liquid extraction and GC-MS. ATS Signature 2 is the elemental profile by ICP-MS [114].

EU focused on methamphetamine (MA) and MDMA between 2004 and 2006 with the research project “Collaborative harmonisation of methods for profiling of amphetamine-type stimulants” (CHAMP), coordinated by the Finish National Bureau of Investigation, resulting in the development of profiling methods for ATS [[191], [192], [193]]. CHAMP studied both the profiling of physical characteristics and the profiling of organic impurities of MDMA tablets. During the project the contribution of diameter, thickness, and weight of tablets to find links between specimens of MDMA was studied. Moreover 43 organic impurities found in MA and 46 organic impurities found in MDMA by GC/MS were considered to allow the selection of the most reproducible target compounds suitable for chemical profiling.

Other analytical methods used in chemical profiling of synthetic illicit drugs of abuse are NMR [194,195], HPLC and capillary electrochromatography [196], capillary electrophoresis [197], and IRMS [198], as reviewed by Onoka et al. for methamphetamine intelligence based on impurity profiles in seizures [199]. Chemical profiles of trace elements in methylamphetamine were studied not only by ICP-MS [200] whereas NAA and PIXE were tested for profiling ecstasy pills [143].

### 5.2 Fentanyl and its analogues

United States is suffering the opioid crisis: 80,000 opioid overdose deaths occurred in 2021, mainly due to fentanyl and its analogues [69]. In about 32 % of deaths, the cause was a mix of fentanyl and stimulants [201].

Fentanyl was synthesized for the first time in 1960 by Paul Janseen and its use as an intravenous anesthetic was approved by the US FDA in 1972 [202]. In 2013 a fentanyl epidemic began in USA, the illicit use of fentanyl increased in the last ten years [203] and reports to the USA National Forensic Laboratory Information System-Drug increased from less than 6.000 in 2014 to more than 100.000 in 2019 [204]. This trend pushed the Drug Enforcement Administration (DEA) to begin in 2017 a systematic study of seized fentanyl samples considering their purity, adulterants, and chemical profiles for intelligence purposes but articles about fentanyl chemical profiles appeared in the scientific literature before 2017. Lurie et al. published a method to analyse 40 fentanyl processing impurities by UHPLC-MS/MS in 2012 [205]. In 2016 Mayer et al. published an article about chemical attribution signatures (CAS) of the analgesic fentanyl. The authors defined CAS as “synthesis precursors and byproducts, impurities, degradation products, and metabolites in various biological matrices” [206]. They studied six synthetic routes to prepare by GC-MS, LC–MS/MS-TOF and ICP-MS and they identified 160 chemical compounds and inorganic species. Statistical treatment partial least-squares-discriminant analysis (PLS-DA) allowed to classify 87 route-specific CAS [207]. Mörén et al. used both GC-MS and UHPLC-HRMS to obtain CAS on many batches of three different fentanyl analogues synthesized in two labs by three chemists, following six different synthetic routes. A multivariate model was used to categorize unknown samples [208]. Ovenden et al. studied two acylation methods used as final step in fentanyl manufacturing and four methods for preparing the fentanyl precursor 4-anilino-*N*-phenethylpiperidine (ANPP) by LC-HRMS and multivariate statistical analysis (MVA) [209]. Toske et al. recently synthesized fentanyl following six different synthetic routes, and samples were analysed by GC-MS, NMR and direct analysis in real-time-mass spectrometry (DART-MS) [210].

Casale et al. analysed carfentanil, which is about 100 times more potent that fentanyl, resulting in a lethal dose of about 20 μg, by IR, NMR, GC-MS, and isotope ratio mass spectrometry [211]. Singleton et al. studied the nitrogen isotope compositions of two intermediates of fentanyl (NPP and ANPP) and developed a GC-C-IRMS method [212].

An interesting development of impurity profiling of synthetic drugs is that it was not only studied with seized bulk materials but also with biological samples such as urine. For example, the impurity alpha-benzyl-N-methylphenethylamine (BNMPA), produced during the synthesis of methamphetamine, was found in two urine specimens out of eighty from drug rehabilitation programs by GC-MS [213]. de Bruin-Hoegée et al. studied the effect of human metabolism on the impurities of fentanyl and identified 23 impurities post-metabolism. They found that it was possible to discriminate between the Gupta and Siegfried synthesis method based on eight impurities in post-metabolism chemical profiles using GC-FID and LC-Orbitrap-MS followed by PCA [214].

The methods used for synthetic illicit drugs profiling are shown in Table 4.

**Table 4:** Synthetic illicit drugs profiling techniques.

Analytical methods	Amphetamines and Amphetamine-type substances (ATS)	Fentanyl and derivatives	References
IR		X	Casale et al., 2017 [211]
TLC	X		Sinnema et al., 1981 [187]
GC-FID	X		Toske et al., 2022 [189]
Capillary electrophoresis	X		Lurie et al., 2004 [197]
GC-MS	X		Sinnema et al., 1981 [187]
	X		Toske et al., 2022 [189]
	X		Collins et al., 2007 [114]
	X		Dujourdy et al., 2008 [191]
	X		Weyermann et al., 2008 [192]
		X	Mayer et al., 2016 [206]
		X	Mörén et al., 2019 [208]
		X	Toske et al., 2023 [210]
		X	Casale et al., 2017 [211]
GC-C-IRMS		X	Singleton et al., 2022 [212]
DART-MS		X	Toske et al., 2023 [210]
HPLC	X		Lurie et al., 2000 [196]
UPLC-Q-TOF		X	Mayer et al., 2016 [206]
UHPLC-MS/MS		X	Lurie et al., 2012 [205]
UHPLC-HRMS		X	Mörén et al., 2019 [208]
LC-HRMS		X	Ovenden et al., 2021 [209]
ICP-MS	X		Toske et al., 2022 [189]
	X		NicDaéid et al., 2013 [200]
		X	Mayer et al., 2016 [206]
GC-IRMS	X		Collins et al., 2015 [198]
NMR	X		Sinnema et al., 1981 [187]
	X		Hays 2005 [194]
	X		Płotka et al., 2012 [195]
		X	Toske et al., 2023 [210]
		X	Casale et al., 2017 [211]
PLS-DA		X	Mayer et al., 2016 [207]
		X	Mayer et al., 2016 [206]

Legend:**IR**: Infra-Red Spectroscopy; **TLC**: Thin-Layer Chromatography; **GC-FID**: Gas Chromatography With Flame-Ionization Detector; **GC-MS:** Gas Chromatography Mass Spectrometry; **HPLC:** High Performance Liquid Chromatography; **UPLC-Q-TOF:** Ultra-High Performance Liquid Chromatography-Quadrupole Time-Of-Flight Mass Spectrometry**; ICP-MS:** Inductively Coupled Plasma Mass Spectrometry; **NMR:** Nuclear Magnetic Resonance; **DART-MS:** direct analysis in real time coupled with mass spectrometry; **HPLC-FID:** High Performance Liquid Chromatography-Flame-Ionization Detector; **GC-IRMS:** Gas Chromatography- Isotope Ratio Mass Spectrometry; **UHPLC-MS/MS:** Ultra-High-Performance Liquid Chromatography-Tandem Mass Spectrometry; **UHPLC-HRMS:** Ultra-High-Performance Liquid Chromatography- High-Resolution Mass Spectrometry; **LC-HRMS:** Liquid Chromatography- High-Resolution Mass Spectrometry; **PLS-DA:** Partial least-squares-discriminant analysis.

## 6 Forensic profiling of other illegal products

The Internet is boosting an illegal market of counterfeit medicines and substandard pharmaceutical products, and the resulting issue is nowadays a global issue because the use of such products can have a serious impact on health, with possible therapeutic failure and acute toxicity [215,216].

One class of medicines often counterfeited are the one to cure erectile dysfunction (e.g., phosphodiesterase type 5 inhibitors). Romolo et al. analysed authentic Viagra® and illegal products containing sildenafil by NAA, LC coupled to HRMS and ion beam analysis (IBA) to obtain chemical profiles [217]. It is very interesting that secondary ion mass spectrometry using ion beams with energies in the range of several MeV (MeV-SIMS) allows both qualitative and quantitative organic analysis [218]. In many occasions it was found on the illicit market that counterfeit benzodiazepine tablets contain novel benzodiazepines and other substances as ingredients not reported on the label [[219], [220], [221], [222]] and/or inaccurate dosages [223]. Profiling of the doping agent oxandrolone was obtained by GC-MS, HPLC-HRMS and three IBA techniques: PIXE, particle induced gamma-ray emission (PIGE) and elastic backscattering spectrometry (EBS) [224]. Piper and Thevis studied two other doping agents (testosterone and boldenone) by carbon isotope ratios of seized [225].

Elemental profiles were studied by Melkegna and Jonah, who analysed traditional herbs used to treat gastrointestinal diseases in Ethiopia by NAA [226].

Serious health issues can be caused not only by counterfeit medicines but also by illegal supplements, such as the ones sold for body weight loss, energy boosting, and erectile dysfunction treatment. Their chemical profiling can be carried out by LC-MS/MS [227].

Pawar et al. published a LC-MS/MS method to obtain the profile of thyroid hormones thyroxine (T4), 3,3′,5-triiodo-l-thyronine (T3), 3,3′,5′-triiodothyronine (rT3), 3,5-diiodothyronine (3,5-T2) and 3,3′-diiodothyronine (3,3′-T2) in dietary supplements [228].

Another global, trans-national criminal issue is food fraud [229] or food crime [230], influencing not only economy but also consumers’ health. Bannor et al. recently published a review about food fraud, affecting especially alcoholic beverages, dairy products, fish, honey, meat, olive oil, milk, spices, wine and coffee, and tea [231]. Analytical techniques used to control food adulteration includes chromatography, NIR, FTIR, Raman spectroscopy, NMR, e-nose/e-tongue, LIBS, MS and Matrix Assisted Laser Desorption Ionization - Time of Flight (MALDI-TOF) Mass Spectrometry [[232], [233], [234], [235], [236]].

Taking as an example coffee, fraudulent strategies include using beans from species of low economic value, mislabelling and the addition of adulterants. Testing for adulterants is not only important for assessing the economic value of the product but also to protect the health of customers. Elemental profiles can allow inferring about the geographical origin of coffee [237]. Among emerging techniques are PIXE [238,239], Fourier Transform Infrared (FTIR), and Accelerator Mass Spectrometry – Radiocarbon Analysis (AMS-^14^C). PIXE allows quantitation of the elements present in the samples, ATR-FTIR probes organic chemical substances and AMS-^14^C provides dating the harvesting time [240]. Chemometrics tools play a key role in food authenticity to manage the large amount of data generated by state-of-the-art analytical methods [241].

The effort to find the source of a chemical substance developed initially for drugs of abuse also entered the scientific area of chemical weapons (CW). The latest development in the field were reviewed by Lu et al., in 2021 [242].

In 1995 a terrorist attack was carried out in Tokyo subway using the nerve agent sarin. The forensic analysis of the CW agent by GC-MS and NMR allowed the identification of three impurities: diisopropyl methylphosphonate (DIMP), diisopropyl phosphorofluoridate (DFP) and hydrogen methylphosphonofluoridate (MPF) corresponding with the synthesis reported in notes seized to the terrorists [243]. In 2010 Fraga et al. studied ten stocks of the nerve-agent precursor methylphosphonic dichloride by LC−MS followed by chemometrics looking for “forensic signatures” [244]. In the same year Hoggard used two dimensional GC × GC/TOF-MS followed by chemometris to study chemical profiles in six samples of dimethyl methylphosphonate (DMMP), a model chemical compound simulating a chemical attack [245]. One year later Fraga et al. wrote another article defining as “chemical forensics” the search of a common source of chemicals (or mixtures) of interest. In this article they use GC-MS to demonstrate for the first time the match between six batches of sarin and an intermediate (methylphosphonic difluoride) based on impurity profiles [246]. In 2016 a paper about “chemical forensics” of the mustard agent tris(2-chloroethyl)amine (HN3) by GC-MS and partial least-squares discriminant analysis (PLSDA) is published [247]. Holmgren et al. studied six different production methods of O-isobutyl S-(2-diethylaminoethyl) methylphosphonothioate (Russian VX). The chemical attribution signatures (CAS) were studied by GC/MS-EI on 37 batches made by two different laboratories [248]. Jansonn et al. studied the chemical profiling of Russian VX in food by liquid LC-MS/MS and multivariate data analysis [249] and Williams et al. developed a chemometric model for the CAS profiles of Russian VX based on both GC-MS and LC-MS analysis [250]. The effect of manufacturing different batches of VX with the same method in different years (2014, 2017 and 2018) was studied by NMR, LC-HRMS, GC-(EI)MS and GC-MS with chemical ionization (CI) [251].

Chemical profiling of CW was carried out also on-site, by both handheld IR and portable Raman. Spectra of 32 preparations obtained by eight different synthetic methods was acquired with no sample preparation, Raman allowed analysis through the walls of sealed glass containers [252].

Due to the importance of CAS in CW investigations, recently the profiling method for the nerve agent precursor methylphosphonic dichloride (DC) was evaluated in eight laboratories by GC-MS, using instruments and methods used in proficiency tests [253].

Matos et al. reviewed in 2019 the state-of-the-art of isotope ratio mass spectrometry to link specimens of Chemical, Biological, Radiological, Nuclear, and Explosives (CBRNE) used in criminal events [254]. A similar review was published in 2022 by Barry et al. who concluded that isotope ratio mass spectrometry shows great potential as a useful tool in forensic application for law enforcement [255]. In Table 5, we summarized the main techniques used for forensic profiling of above-mentioned illegal products.

**Table 5:** Other illegal products forensic profiling.

Analytical methods	Phosphodiesterase type 5 inhibitors	BNZs	Doping agent	Medicinal plants	Food supplements	Food	Pesticides	CW agents	References
IR							X	X	Lu et al., 2021 [242]
									Wiktelius et al., 2018 [252]
NIR						X			Aslam et al., 2023 [234]
GC-MS		X							May et al., 2020 [220]
	X		X						Mestria et al., 2022 [224]
		X							Blakey et al., 2022 [223]
		X							Downey et al., 2022 [221]
						X			Nolvachai et al., 2023 [232]
						X			Suman et al., 2021 [236]
							X	X	Lu et al., 2021 [242]
								X	Miyata et al., 2000 [243]
								X	Fraga et al., 2011 [246]
								X	Fraga et al., 2016 [247]
								X	Williams et al., 2018 [250]
								X	Ovenden et al., 2020 [251]
								X	Holmgren et al., 2023 [253]
								X	Holmgren et al., 2018 [248]
								X	Ovenden et al., 2020 [251]
MALDI-TOF						X			Zambonin 2021 [235]
FTIR						X			Aslam et al., 2023 [234]
						X			Chytry et al., 2022 [240]
LC-MS		X							May et al., 2020 [220]
				X					Esposito et al., 2023 [227]
					X				Esposito et al., 2023 [227]
					X				Pawar et al., 2022 [228]
							X	X	Lu et al., 2021 [242]
								X	Fraga et al., 2010 [244]
								X	Jansson et al., 2018 [249]
								X	Williams et al., 2018 [250]
LC-HRMS	X								Romolo et al., 2019 [217]
								X	Ovenden et al., 2020 [251]
HPLC-HRMS	X		X						Mestria et al., 2022 [224]
GC-TOF								X	Hoggard et al., 2010 [245]
NMR						X			Sobolev et al., 2019 [233]
						X			Aslam et al., 2023 [234]
							X	X	Lu et al., 2021 [242]
								X	Miyata et al., 2000 [243]
								X	Ovenden et al., 2020 [251]
NAA	X								Romolo et al., 2019 [217]
	X								Romolo et al., 2021 [218]
				X					Melkegna et al., 2021 [226]
IBA	X								Romolo et al., 2019 [217]
	X								Romolo et al., 2021 [218]
	X		X						Mestria et al., 2022 [224]
EBS	X		X						Mestria et al., 2022 [224]
CIR			X						Piper et al., 2022 [225]
LIBS						X			Aslam et al., 2023 [234]
Raman spectroscopy						X			Aslam et al., 2023 [234]
								X	Wiktelius et al., 2018 [252]
PIXE						X			Debastiani et al., 2019 [238]
						X			Cloete et al., 2019 [239]
						X			Chytry et al., 2022 [240]
AMS						X			Chytry et al., 2022 [240]
Chemometrics tools						X			González-Domínguez et al., 2022 [241]
IRMS								X	Matos et al., 2019 [254]
								X	Barry et al., 2022 [255]

Legend:**BNZs:** Benzodiazepines; **IR**: Infra-Red Spectroscopy; **IRMS**: Isotope Ratio Mass Spectrometry; **GC-MS:** Gas Chromatography Mass Spectrometry; **HPLC-HRMS:** High Performance Liquid Chromatography/High-Resolution Mass Spectrometry; **LC-HRMS:** Liquid Chromatography-High-Resolution Mass Spectrometry; **LC-MS:** Liquid Chromatography Mass Spectrometry; **NMR:** Nuclear Magnetic Resonance; **NAA**: Neutron Activation Analysis; **PIXE**: Proton Induced X-Ray Emission; CIR: carbon isotope ratios; MALDI-TOF: Matrix assisted laser desorption/ionization-Quadrupole Time-Of-Flight Mass Spectrometry; **FTIR:** fourier transform spectroscopy; **NIR Spectrophotometer:** near-infrared spectrophotometer; **LIBS:** laser induced breakdown spectroscopy; **AMS:** accelerator mass spectrometry; **EBS:** elastic backscattering spectrometry; **GC-TOF:** Gas Chromatography- Quadrupole Time-Of-Flight Mass Spectrometry: **GC-MS-EI:** Gas Chromatography- Mass Spectrometry-electronic ionization: **IB**.

**BNZs:** Benzodiazepines; **IR**: Infra-Red Spectroscopy; **IRMS**: Isotope Ratio Mass Spectrometry; **GC-MS:** Gas Chromatography Mass Spectrometry; **HPLC-HRMS:** High Performance Liquid Chromatography/High-Resolution Mass Spectrometry; **LC-HRMS:** Liquid Chromatography-High-Resolution Mass Spectrometry; **LC-MS:** Liquid Chromatography Mass Spectrometry; **NMR:** Nuclear Magnetic Resonance; **NAA**: Neutron Activation Analysis; **PIXE**: Proton Induced X-Ray Emission; **CIR:** carbon isotope ratios; **MALDI-TOF:** Matrix assisted laser desorption/ionization-Quadrupole Time-Of-Flight Mass Spectrometry; **FTIR:** fourier transform spectroscopy; **NIR Spectrophotometer:** near-infrared spectrophotometer; **LIBS:** laser induced breakdown spectroscopy; **AMS:** accelerator mass spectrometry; **EBS:** elastic backscattering spectrometry; **GC-TOF:** Gas Chromatography- Quadrupole Time-Of-Flight Mass Spectrometry: **GC-MS-EI:** Gas Chromatography- Mass Spectrometry-electronic ionization: **IB**.

## 7 Forensic profiling of explosives

Forensic explosives investigations include the reconstruction of the production and origin of energetic materials found in the charge(s) of an improvised explosive device (IED) at the crime scene.

In 1975 Nissenbaum published an article about stable isotopes of carbon in trinitrotoluene (TNT), the most important explosive in blasting charges [256]. The natural variations from different sources, hardly affected after the manufacturing of the explosives, showed to be a possible tool for the differentiation of specimens [257]. McGuire et al. collected results of analysis of stable isotope ratios after detonating charges containing HMX, TNT, or triamino trinitrobenzene (TATB) in explosion tests with PETN boosters during research carried out by the Lawrence Livermore National Laboratory for the U.S. Department of Energy [258].

The study of stable isotopes is not the only approach to obtain chemical profiles of explosives. Brust et al. used a GC–MS method for the identification and quantification of impurities in TNT to investigate relations between TNT specimens encountered in forensic casework using likelihood ratios (LRs) [259] (Table 6).

**Table 6:** Techniques employed for explosives profiling.

Analytical methods	Isotope ratio	Impurities	TNT	Hexamine and RDX	AN	Trace elemental composition	ANFO	PETN	Semtex	TATP	HMTD	UN	ETN	References
GC-MS		X												Brust et al., 2014 [259]
							X							Suppajariyawat et al., 2019 [270]
HPLC			X											Coffin et al., 2001 [260]
								X						Howa et al., 2014 [279]
				X				X						Howa et al., 2016 [280]
HPLC-MS		X						X						Brown et al., 2014 [274]
LC-MS		X						X						Brust et al., 2013 [276]
		X						X						Brust et al., 2014 [275]
													X	Bezemer et al., 2020 [288]
GC/ITMS/IRMS	X													Coffin et al., 2001 [260]
IRMS	X													Phillips et al., 2003 [261]
	X			X										Lock et al., 2008 [263]
					X									Benson et al., 2009 [266]
					X									Benson et al., 2010 [265]
					X									Brust et al., 2015 [268]
					X									Hu et al., 2021 [269]
			X	X	X			X						Chesson et al., 2016 [281]
								X		X				Benson et al., 2009 [284]
												X		Aranda et al., 2011 [286]
											X			Lock et al., 2012 [285]
													X	Bezemer et al., 2020 [289]
EA/Conflo/IRMS					X									Howa et al., 2014 [267]
			X				X	X						Widory et al., 2009 [271]
								X						Howa et al., 2014 [279]
				X				X						Howa et al., 2016 [280]
									X					Pierrini et al., 2007 [282]
ICP-MS						X								Brust et al., 2015 [268]
												X		D'Uva et al., 2020 [290]
FTIR							X							Suppajariyawat et al., 2019 [270]
				X				X						Howa et al., 2016 [280]
												X		D'Uva et al., 2022 [273]
HS-GC-FID							X							Garzón-Serrano et al., 2020 [272]
GC-ECD			X											Jenkins et al., 2001 [278]
Raman Spectroscopy												X		D'Uva et al., 2021 [287]
FESEM												X		D'Uva et al., 2021 [287]
IC												X		D'Uva et al., 2021 [287]
GCxGC-TOF-MS			X	X				X						Stefanuto et al., 2015 [277]

Legend:**TNT:** Trinitrotoluene**; RDX:** Cyclotrimethylenetrinitramine also known as Royal Demolition eXplosive; **AN:** Ammonium Nitrate; **ANFO:** ammonium nitrate fuel oil; **PETN**: Pentaerythritol Tetranitrate; **TAPT:** Triacetone Triperoxide; **HMTD: UN:** Urea Nitrate; ETN: **GC-MS:** Gas Chromatography Mass Spectrometry; **HPLC:** High Performance Liquid Chromatography; **HPLC-MS:** High-Performance Liquid Chromatography-Mass Spectrometry; **LC-MS:** Liquid Chromatography-Mass Spectrometry; **GC/ITMS/IRMS:** Gas Chromatography/Ion Trap Mass Spectrometry/Isotope Ratio Mass Spectrometry; **IRMS**: Isotope Ratio Mass Spectrometry; **EA/Conflo/IRMS:** Elemental Analyzer interfaced to an isotope ratio mass spectrometer via Continuous-Flow interface; **ICP-MS:** Inductively Coupled Plasma-Mass Spectrometry; **FTIR:** Fourier Transform Infrared Spectroscopy; **HS-GC-FID:** Headspace-Gas Chromatography with flame ionization detection; **GC-ECD:** Gas Chromatography-Electron Capture Detector; **FESEM:** Field Emission Scanning Electron Microscope; **IC:** Ion Chromatography; **GCxGC-TOF-MS:** Two-Dimensional Gas Chromatography combined with Time-Of-Flight Mass Spectrometry.

Nitroaromatics are common contaminants at explosives production and testing sites. They can move through groundwater for miles, resulting in complex ecosystems needing where sources of pollution need to be identified. Coffin et al. compared d^13^C and d^15^N for five different sources of TNT to study the spatial variation in groundwaters [260]. One year after this publication, in 2002, the University of Reading and the Forensic Explosives Laboratory (FEL), United Kingdom Defence Science and Technology Laboratory (DSTL) formed the Forensic Isotope Ratio Mass Spectrometry (FIRMS) Network and a conference was held on 16 and 17 September 2002. The isotopic analysis of samples of ammonium nitrate (AN), blackpowder, flashpowders, chlorate and perchlorate mixtures, sodium chlorate weedkiller, nitrocellulose, nitromethane, sugar, smokeless powder, TNT, nitroglycerine (NG), pentaerythritol tetranitrate (PETN), cyclotrimethylenetrinitramine (RDX), cyclotetramethylene tetranitramine (HMX), triacetone triperoxide (TATP), plastic explosive no. 4 (PE4) showed that the technique had the potential for the forensic analysis of explosives [261,262].

A specific reason for forensic profiling of explosives is the search of links between an explosive precursor and its product as part of the criminal investigation. Lock and Meier-Augenstein studied the high explosive RDX and the isotopic links with its precursor hexamethylenetetramine (hexamine) [263]. RDX poses environmental issues as nitroaromatics and a method based on δ^15^N and δ^18^O was developed and applied to RDX samples after to biodegradation under aerobic or anaerobic conditions by Bernstein et al., to monitor RDX contamination in the environment [264].

A precursor particularly important in the manufacturing of explosives is AN, used as major component in terrorist attacks carried out in UK in the nineties, in the Oklahoma City bombing in 1995 and in Oslo bombing in 2011. Benson et al. validated data for the measurement of nitrogen isotope ratios in AN [265], development of a preliminary AN classification scheme and was able to differentiate AN prills from three different Australian manufacturers combining oxygen and hydrogen stable isotope values and specimens from five different overseas sources using a combination of the nitrogen, oxygen and hydrogen isotope values [266]. Another approach based on sodium tetraphenylborate showed that ammonium nitrogen and nitrate nitrogen in AN have independent sources of isotopic variation [267].

Brust et al. published later their research about isotopic and elemental profiling to discriminate between different batches of AN and calculated likelihood ratios using linear discriminant analysis [268].

Hu et al. developed a procedure to purify AN prior to IRMS analysis and discriminated AN specimens from eight different cities in China [269].

The explosive ammonium nitrate fuel oil (ANFO) contains about 95 % AN and about 5 % liquid hydrocarbons (fuel oil). Suppajariyawat et al. analysed eight different ANFOs prepared with eight different diesel brands collected in UK in two seasons (winter and summer) by GC–MS and FTIR. GC–MS found four fatty acid methyl ester (FAME) in all summer samples but did not find in some winter samples. Pre-blast ANFO samples analytical results were studied by principal component analysis (PCA) and Lineal Discriminant Analysis (LDA), allowing high classification performance [270].

Widory et al. differentiated ANFO, PETN and TNT specimens by both their specific isotope signatures obtained by a multi-stable isotope (δ13C, δ15N, δ18O, δD) approach and their combination with corresponding element concentrations [271].

Garzón-Serrano et al. analysed ammonia and other volatile compounds from seized ANFO specimens using a HS-SPME/GC-FID methodology and demonstrated that the combination of results from GC analysis with scanning electron microscopy (SEM) allows the differentiation between ANFO with different origins [272].

D'Uva et al. used ATR-FTIR spectroscopy, trace elemental analysis and chemometrics to determine the sour e of homemade AN [273].

PETN is one of the most powerful explosives, used in both military and civilian applications. Brown and Giambra studied the impurities of PETN by HPLC-MS and showed that batches can be differentiated based on relative concentration profiles [274]. Brust et al. also studied post-explosion PETN samples by LC–MS and found that the impurity profile can be used to investigate the relationship between a suspect and an explosion site in casework [275,276].

The application of GC × GC-TOF-MS analysis for headspace profiling of PETN, RDX, and TNT was published by Stefanuto et al., who compared the use of different sampling methods with SPME and different chromatographic analysis. Statistical work on complex chromatograms obtained by fast GC × GC was studied to discriminate different commercial explosives. According to the authors, this type of research about profiling headspace of explosives is also useful for dog training [277]. Jenkins et al. studied headspace at equilibrium of military-grade explosives and land mine signature chemicals by SPME and electron capture detector (GC-ECD) [278].

Howa et al. studied carbon (δ13C) and nitrogen (δ15N) isotope ratios in reactants, pentaerythritol (PE) and nitric acid, and 175 PETN samples from 22 manufacturing facilities. They showed that δ13C values of PETN were indistinguishable from that of the reactant pentaerythritol and δ15N variation in PETN depends on both nitric acid δ15N and conditions of reaction. Authors were also able to discriminate explosive blocks from the same manufacturer [279].

Both RDX and PETN are used with binders and other chemical substances in plastic-bonded explosives (PBX). Howa et al. analysed extracts from PBXs and identified plasticizers, oils, dyes, and antioxidants by GC and explosive chemical compounds by HPLC-UV. The stable isotope ratios of isolated components were also studied, allowing the discrimination between two specimens of PBX containing RDX (called C-4) identical based on traditional qualitative and quantitative chemical analysis [280]. C-4 was studied also by Chesson et al., who extended their research to another PBX called Semtex, containing the explosive chemical compound PETN and RDX. The research focused on preparing different chemical compounds for stable isotope analysis and applying optimised solvent extraction methods to explosive specimens possibly containing RDX, HMX, PETN, TNT, AN, nitrocellulose, and other additives [281].

Pierrini et al. proposed a likelihood ratio approach to specifically evaluate stable isotope data acquired from semtex samples [282].

In the last decades the terrorist attacks involving improvised explosives (IE) or homemade explosives (HME) became more and more common. IE are manufactured by criminals using readily available materials. One of them is triacetone triperoxide (TATP), a very dangerous organic peroxide, highly sensitive to impact, friction and heating [283]. Benson et al. successfully measured carbon, hydrogen and oxygen isotope composition in 14 TATP samples and discriminating TATP samples from different sources based on 3D plot. They also studied carbon and nitrogen isotope values in PETN samples [284].

Another peroxide used as IE is hexamethylene triperoxide diamine (HMTD). Lock et al. prepared HMTD samples using various precursors and different experimental conditions and found that results are affected by changes of the concentration of hydrogen peroxide [285].

Another IE is urea nitrate (UN), made of uronium and nitrate ions. Aranda et al. developed a method to separate UN into its component ions for δ15N measurements and proposed an approach useful to discriminate between UN specimens otherwise identical from the chemical point of view [286].

D'Uva et al. reported for the first time the synthesis and characterisation of UN from retail products easily accessed. Raman was able to identify UN in an unknown specimen but did not reveal any observable differences between samples prepared during the research. IR could both easily identify UN and discriminate some samples, based on a peak at 1150 cm^−1^. FESEM-EDS could confirm whether UN has been prepared from an Osmocote product based on morphology and elemental composition. IC was also useful to distinguish the Osmocote samples from other sources of UN. Finally, ICP-MS showed the greatest discriminatory power, based on the concentration of nine elements present at varying concentrations [287].

Another IE is erythritol tetranitrate (ETN), studied by Bezemer et al. by LC-MS with the aim of providing forensic information about the production and origin of explosive found in casework [288].

In a follow-up study IRMS analysis was carried out to predict possible links between ETN samples and its precursors based on carbon, nitrogen, hydrogen and oxygen isotope ratios. Carbon and nitrogen from erythritol and nitric acid or a nitrate salt used as precursors are the only source of atoms in ETN, resulting in robust linear relationships observed. According to the authors, the combination of LC–MS with IRMS results is expected to increase the robustness of the forensic comparison of ETN specimens in forensic casework [289].

Pyrotechnic accessible and inexpensive in Australia can be used to prepare IE. D'Uva et al. used ICP-MS to analyse 50 elements in 48 pre-blast sparkler samples. PCA was used to examine analytical results and LDA allowed the development of a discriminant model, which demonstrated to successfully classify 100 % of the samples to their correct brand [290]. Flash bangers seized by the Dutch police were studied by Bezemer et al., who found that differentiation is possible in many cases based on visual examinations. When visual features are lost, characterization can be carried out by in depth chemical or physical analysis [291]. Profiling approaches are not limited to the explosive components of a charge but can be applied also to other components of the ordnance or IED. The plastic caps of flash bangers were studied by XRF and LA-ICP-MS to obtain their elemental profiles. The authors concluded that visual examination combined with XRF was the optimal approach to compare intact caps [292].

## 8 Discussion and conclusions

Forensic profiling or chemical attribution signatures (CAS) has a long history, beginning in 1948 with opium. At that time the aim was to infer about the origin of the seized material. In the following decades, this approach was used for different illegal substances with a double aim: strategic intelligence and tactical intelligence. The former is about the geographical origin of a specimen. The latter involve the comparison of two or more samples, aiming to infer about a common origin [201,293]. A first step in tactical intelligence is the physical profiling, i.e., the thorough forensic characterization of features such as shape and size of seized material (e.g., tablets or blocks) and packaging materials (e.g., plastic bags, plastic films, adhesive tapes). The second step in tactical intelligence, which is also necessary in strategic intelligence, is the study of the chemical profiles.

The amount and variety of drugs on the illegal market are increasing but seizures, sometimes of very large amount, are also increasing and police operations disrupting criminal organizations involved in drug trafficking never stop. Different intelligence tools, including forensic profiling, support police activities. This review showed that in the last years the class of products subjected to analysis to obtain CAS are increasing, not only considering new drugs of abuse, such as fentanyl derivatives, but also new substances such as chemical warfare agents and explosives, and new products such as medicines, supplements, and food.

There are three main reasons to support forensic profiling.

a. health and safety,
b. reliable and relevant features in this drug intelligence perspective (e.g., CHAMP project),
c. precursors to be controlled.

Forensic profiling can provide early warning for toxic chemical substances in illegal products made available by criminals in a specific territory at a given time. The EU research project CHAMP demonstrated that forensic profiling is useful to establish the synthetic routes of ATS and possibly to find connections between drug seizures. Establishing the production of drugs of abuse following a new synthetic route, allows authorities to include new chemical compounds among the precursors to be controlled.

Chromatographic techniques coupled to mass spectrometry are among the main analytical tools useful to collect organic profiles due to its robustness, reproducibility, sensitivity and speed. GC-MS technique is widely used for the analysis of drugs of abuse, although FID was often used in several profiling studies. Electron impact ionization (EI) is the most commonly used ionization technique profiling, while others, i.e., chemical ionization (CI) in positive/negative mode. LC coupled to atmospheric pressure ionization (API) are preferred in specific forensic analysis such as phytocannabinoids in biological samples. Several authors preferred LC over GC also in cannabinoid profiling due to the simplified sample preparation methods, along with avoidance of analytes loss.

Another very important approach to forensic profiling is the one based on techniques allowing on-site analysis, such as Raman, FTIR and NIR. Especially handheld FTIR and NIR instruments showed their capability to provide timely, rapid, versatile and non-invasive qualitative and quantitative profiling where the material is found, especially when chemometrics is used to exploit the collected data.

Raman, FTIR and NIR are also used to evaluate the growth staging and curing process of cannabis plant. Elemental profiles were obtained by NAA before the advent of ICP-MS. The most recent advances in the field are IRMS, useful both in strategic and tactical intelligence, and IBA. MeV-SIMS, belonging to the family of IBA approaches, allows both elemental profiles and organic analysis. According to the reviews by Matos et al. [254] and Barry et al. [255], IRMS of CBRNE materials is a promising technique for forensic investigations and worth of continued research and funding. IBA has the great advantage of giving the opportunity to collect both elemental and organic profiles.

Another interesting development of CAS is the analysis of biological samples. de Bruin-Hoegée et al. recently demonstrated that it is possible to discriminate between the Gupta and Siegfried synthesis of fentanyl based on post-metabolism impurity profiles [214].

To ensure an effective support to police activities, law enforcement agencies need to work in close collaboration with forensic laboratories not only to address issues such as financial resources [294], but also to develop the most fruitful forensic profiling approach to deal with the latest threat for the health and security of citizens worldwide.

After 75 years forensic profiling is not limited to few drugs of abuse as it happened only a couple of decades ago but showed the possibility to provide information related to new drugs of abuse present on illegal markets, such as fentanyl derivatives, or other classes of substances, such as illegal medicines, doping agents, supplements, food, chemical warfare agents (CWA) and explosives. The use of forensic profiling with new classes of substances opened new research areas, such as the novel benzodiazepines and other substances used as ingredients but not reported on the label of counterfeit benzodiazepine tablets found on the illicit market.

The future of forensic profiling is challenging as challenging is the dynamic nature of both the criminal organizations and the illegal markets. Organized crime is involved in the manufacturing of more and more illegal products and forensic profiling is a very powerful tool to support the health of citizens and the administration of justice worldwide. To maintain the capabilities of profiling products already studied and to allow the study of new classes of illegal products a strong collaboration between LEAs and scientific institutions is needed. Analytical power is useless without effective exchange of information with LEAs and among LEAs, possibly based on common databases. Future research is needed not only on new classes of illegal products and drugs metabolites, but also on new analytical techniques, providing more rapid analysis. Development in chemometrics is also desirable to look for “forensic signatures” useful to establish authenticity of products and to compare and interpret large amount of forensic data. Another promising area of future research is the use of combinations of different analytical techniques. This multi-techniques approach implies the need of advanced data fusion capabilities, to provide valuable forensic information to law enforcement based on data obtained from different analytical approaches.

It is finally very important the selection of the best approach to use profiling results from casework in court. Pierrini et al. proposed in 2007 a likelihood ratio approach to evaluate stable isotope data from semtex samples and concluded that international collaboration is essential for establishing a significant database useful for counter-terrorism [282]. Bolck et al., in 2009 compared two likelihood ratio (LR) approaches to evaluate the strength of evidence of analytical results from MDMA tablets [295]. Brust et al. calculated later the evidential value of impurity profiles of TNT samples using likelihood ratios based on analytical results obtained by vacuum-outlet GC–MS [259]. Likelihood ratios were also calculated by combining elemental and isotopic profiling of AN samples to discriminate their sources [268]. Recently Vangerven et al. published a paper about impurity profiling of metabolized samples to find markers specific of the synthetic route and used LDA to establish likelihood ratios (LRs) [296]. These articles are not a majority of the references considered in this review and there is a debate in scientific literature on using likelihood ratios to indicate the evidential strength. Among the different views, Oaksford et al. consider conclusions based on a likelihood ratio useful to respect the role of the expert for the evaluation of the evidence and to avoid fallacious reasoning and errors which could put a defendant at risk [297]. Moreover, for Vuille et al. conclusions based on a likelihood ratio satisfy the right to a fair trial [298].

Regardless of the use of the results of for police intelligence or as evidence in court, forensic profiling, after 75 years, is still a very powerful tool to support the health of citizens and the administration of justice worldwide and we hope that this review will support the activities of scientists to develop improved approaches in this field, deserving multiple disciplinary contributions.

## CRediT authorship contribution statement

**Roberta Tittarelli:** Writing – original draft, Investigation. **Sara Dagoli:** Writing – original draft, Investigation. **Rossana Cecchi:** Writing – review & editing, Supervision, Methodology, Conceptualization. **Luigi Tonino Marsella:** Writing – review & editing, Supervision, Methodology, Conceptualization. **Francesco Saverio Romolo:** Writing – original draft, Supervision, Project administration, Methodology, Investigation, Conceptualization.

## Data availability statement

No data was used for the research described in the article.

## Declaration of competing interest

The authors declare that they have no known competing financial interests or personal relationships that could have appeared to influence the work reported in this paper.
